# Distributed Regression Analysis Application in Large Distributed Data Networks: Analysis of Precision and Operational Performance

**DOI:** 10.2196/15073

**Published:** 2020-06-04

**Authors:** Qoua Her, Jessica Malenfant, Zilu Zhang, Yury Vilk, Jessica Young, David Tabano, Jack Hamilton, Ron Johnson, Marsha Raebel, Denise Boudreau, Sengwee Toh

**Affiliations:** 1 Harvard Medical School Harvard Pilgrim Health Care Institute Boston, MA United States; 2 Institute for Health Research Kaiser Permanente Colorado Denver, CO United States; 3 Center for Observational Research and Data Science Bristol-Meyers Squibb Lawrenceville, NJ United States; 4 Division of Research Kaiser Permanete North California Oakland, CA United States; 5 Health Research Institute Kaiser Permanente Washington Seattle, WA United States

**Keywords:** distributed regression analysis, distributed data networks, privacy-protecting analytics, pharmacoepidemiology, PopMedNet

## Abstract

**Background:**

A distributed data network approach combined with distributed regression analysis (DRA) can reduce the risk of disclosing sensitive individual and institutional information in multicenter studies. However, software that facilitates large-scale and efficient implementation of DRA is limited.

**Objective:**

This study aimed to assess the precision and operational performance of a DRA application comprising a SAS-based DRA package and a file transfer workflow developed within the open-source distributed networking software PopMedNet in a horizontally partitioned distributed data network.

**Methods:**

We executed the SAS-based DRA package to perform distributed linear, logistic, and Cox proportional hazards regression analysis on a real-world test case with 3 data partners. We used PopMedNet to iteratively and automatically transfer highly summarized information between the data partners and the analysis center. We compared the DRA results with the results from standard SAS procedures executed on the pooled individual-level dataset to evaluate the precision of the SAS-based DRA package. We computed the execution time of each step in the workflow to evaluate the operational performance of the PopMedNet-driven file transfer workflow.

**Results:**

All DRA results were precise (<10^−12^), and DRA model fit curves were identical or similar to those obtained from the corresponding pooled individual-level data analyses. All regression models required less than 20 min for full end-to-end execution.

**Conclusions:**

We integrated a SAS-based DRA package with PopMedNet and successfully tested the new capability within an active distributed data network. The study demonstrated the validity and feasibility of using DRA to enable more privacy-protecting analysis in multicenter studies.

## Introduction

### Background and Significance

**Figure 1:**
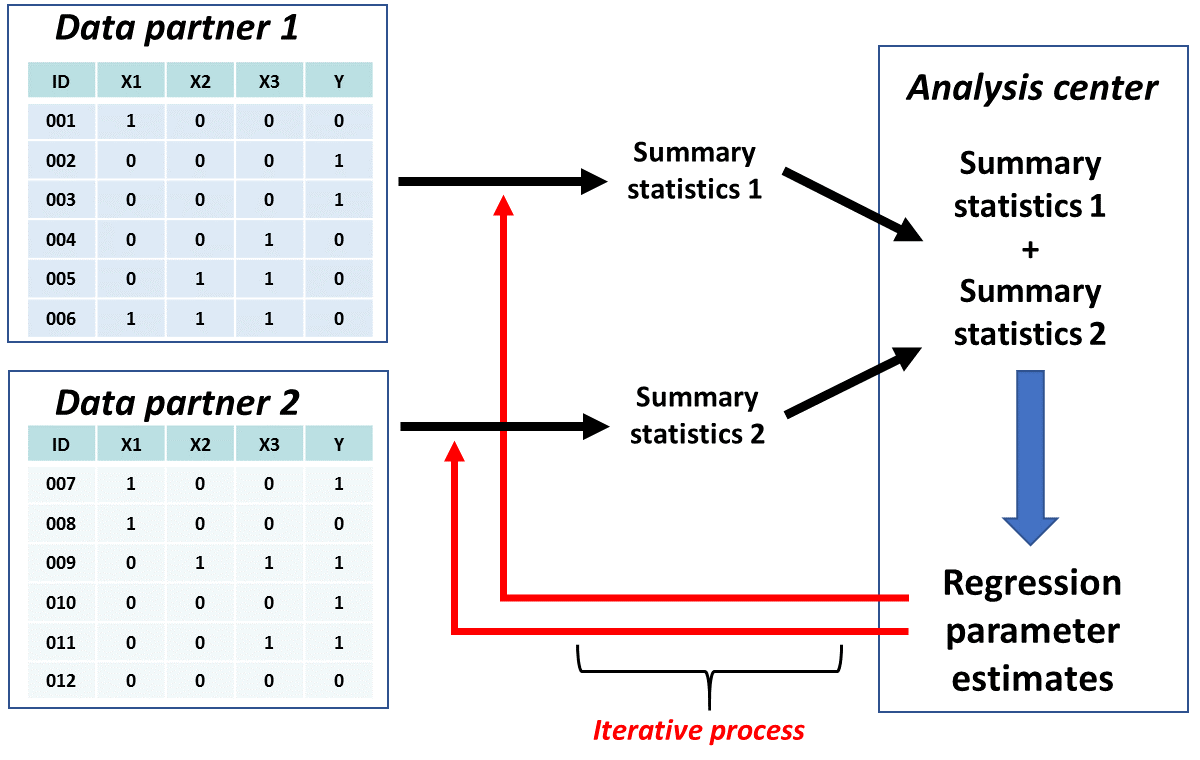
Distributed regression analysis with horizontally partitioned data.

Distributed regression analysis (DRA) is a suite of methods that perform multivariable regression analysis in multicenter studies without the need for pooling individual-level data [1,2]. Data partners compute highly summarized intermediate statistics (eg, sums of squares and cross products matrices) of their individual-level data and share these statistics with a trusted third-party or analysis center (Figure 1). The analysis center aggregates the intermediate statistics, assesses model convergence, and computes the regression parameter estimates. DRA is mathematically equivalent to the conventional regression analysis of pooled individual-level data. It achieves the same level of statistical sophistication using only summary-level information, thereby offering better protection for individual and institutional privacy without jeopardizing the scientific rigor of the analysis.

However, DRA is not widely used in practice due to the operational challenges in implementing the approach [3]. The modeling process of common regression analyses (eg, logistic regression, Cox proportional hazards regression) is iterative and requires multiple exchanges of highly summarized intermediate statistics between the data partners and the analysis center. Manual execution of DRA is labor-intensive and highly susceptible to human errors (eg, transfer of incorrect files). There have been efforts to develop capabilities that coordinate and automate the iterative computation and file transfer process of DRA to make it a more practical analytical option in real-world multicenter studies [4-11]. These efforts have focused primarily on the programming language R and specially designed applications (eg, Java applets) to facilitate semiautomated or fully automated file transfers between the data partners and the analysis center [7-11]. The performance of these capabilities has largely been tested in simulated or relatively well-controlled environments [4-8], and no DRA application has been developed in SAS, another commonly used statistical software.

In our previous work, we enhanced PopMedNet, an open-source distributed networking software currently used by several large national distributed data networks (DDNs), to enable an automatable and iterative file transfer workflow for routine implementation of DRA [3]. This workflow coordinates and automates the iterative transfer of files between the data partners and the analysis center. We also created a SAS-based DRA package to conduct distributed linear, logistic, and Cox proportional hazards regression analysis in horizontally partitioned DDN [12,13], environments where each data partner holds information about distinct individuals [14,15]. We integrated the PopMedNet workflow with the SAS-based DRA package to create a DRA application.

### Objectives

Despite the appealing theoretical properties of DRA, applications designed to perform the analysis can still be inoperable or produce biased results in real-world settings due to unappreciated factors (eg, human errors in execution, incompatible or different software versions, network or firewall restrictions, and network conditions). Evaluating the precision of DRA applications compared with the pooled individual-level data analysis and the feasibility of performing the analysis in reasonable execution times in real-world settings is needed to demonstrate DRA as a practical and valid analytical method. In this study, we demonstrate the feasibility of using the SAS-based DRA package and PopMedNet-driven file transfer workflow to perform DRA in a real-world horizontally partitioned DDN. Specifically, we quantify the precision of the SAS-based DRA package and the operational performance of the PopMedNet-driven file transfer workflow.

## Methods

### Study Setting: The Sentinel System

Funded by the US Food and Drug Administration, the Sentinel System is an active surveillance system designed to monitor the safety of approved medical products using longitudinal, regularly updated electronic health data from a network of 18 health plans and health care delivery systems [16,17]. Sentinel data partners transform their data into a common data model [18], which enables analytical programs and tools to be centrally developed and executed across data partners with minimal modifications. Over the years, the system has developed a suite of version-controlled, customizable, and freely available modular programs to rapidly query the transformed data across the DDN [19]. Among the tools is the Cohort Identification and Descriptive Analysis (CIDA) tool, a SAS program that assembles cohorts of individuals according to user-specified study parameters (eg, exposures, outcomes, inclusion and exclusion criteria) using established coding systems (eg, International Classification of Diseases, Ninth or Tenth Revision, Clinical Modification; National Drug Codes). The CIDA tool can generate a harmonized (ie, with the same covariates and covariate names) individual-level dataset at each data partner. Users can employ other tools (eg, Propensity Score Analysis Tool) or develop ad hoc analytical programs to query these datasets behind the data partner’s firewall for complex inferential analyses.

Sentinel uses PopMedNet to facilitate file transfers between the data partners and the Sentinel Operations Center [20]. The Sentinel Operations Center, which serves as the analysis center for all Sentinel queries, uses a Web-based portal to create and securely distribute queries to data partners via PopMedNet. The data partners use a locally installed Microsoft Windows application, known as the DataMart Client, to retrieve the query and return the requested dataset, usually in aggregate-level format, to the Sentinel Operations Center. All file transfers between data partners and the Sentinel Operations Center are accomplished through secure HTTPS, secure sockets layer, or transport layer security connections. PopMedNet security and authentication requirements ensure that only approved queries are submitted to and responses returned by prespecified and approved data partners. In addition, the PopMedNet workflow is agnostic to query types, file formats (RData, sas, .docx, etc) and can transfer individual file sizes up to 2 GB.

### SAS-Based Distributed Regression Analysis Application

There are numerous algorithms (eg, secure data integration, secure summation) for DRA in horizontally partitioned DDNs, environments where each data partner holds information about distinct patient cohorts [21,22]. In our previous work, we created a SAS-based DRA package comprising 2 interlinked SAS packages (one executed at the data partners and the other at the analysis center) using 2 algorithms: (1) distributed iteratively reweighted least squares to perform distributed linear and logistic regression analysis [12], and (2) distributed Newton-Raphson algorithm to perform distributed Cox proportional hazards regression analysis using the Efron or Breslow approximation for tied event times [13]. Both algorithms utilize a semitrusted third-party as the analysis center to aggregate the highly summarized intermediate statistics (eg, sums of squares and cross products matrices) and compute regression parameter estimates and SEs. We define a semitrusted third-party as a party that data partners trust with their summary-level information but not with their individual-level data. This party does not share data from any data partner with other data partners without consent, does not attempt to derive the individual-level data from the intermediate statistics, does not collude with data partners to derive any information about other data partners’ individual-level data, and follows the DRA algorithms [23].

We provide a brief overview of the distributed iteratively reweighted least squares and the Newton-Raphson algorithms used to implement the SAS-based DRA package for distributed linear, logistic, and Cox proportional hazards regression analysis using the Sentinel Operations Center as the analysis center in Multimedia Appendix 1. A detailed description of these algorithms is available elsewhere [12,13].

### PopMedNet Enhancements to Enable Automatable Distributed Regression Analysis

Both the distributed iteratively reweighted least squares and Newton-Raphson algorithms in the SAS-based DRA package utilize a master-worker process, where the analysis center directs the iterative DRA computations and the data partners execute these computations on their individual-level data with input (eg, updated regression parameter estimates) from the analysis center. Thus, an iterative file transfer workflow is required to transfer the highly summarized intermediate statistics and the updated regression parameter estimates between the data partners and the analysis center until the model converges or the analysis reaches a prespecified maximum number of iterations.

We previously enhanced PopMedNet to create an iterative and automatable file transfer workflow to facilitate routine DRA [3]. In brief, we built a back-end component, referred to as the *DRA-adapter,* into PopMedNet to allow the DataMart Client to upload files automatically and iteratively from and download files to prespecified folders at the data partners and the analysis center. We also developed functionalities for folder monitoring and trigger file creation and deletion in the DataMart Client to integrate the PopMedNet workflow with the two interlinked SAS packages of our SAS-based DRA package. A full description of the PopMedNet workflow and its integration with the SAS-based DRA package is available elsewhere [12,13]. We collectively refer to the integration of the SAS-based DRA package and the PopMedNet-driven file transfer workflow as the DRA application hereafter.

### Distributed Regression Analysis: A 3-Step Process

**Figure 2:**
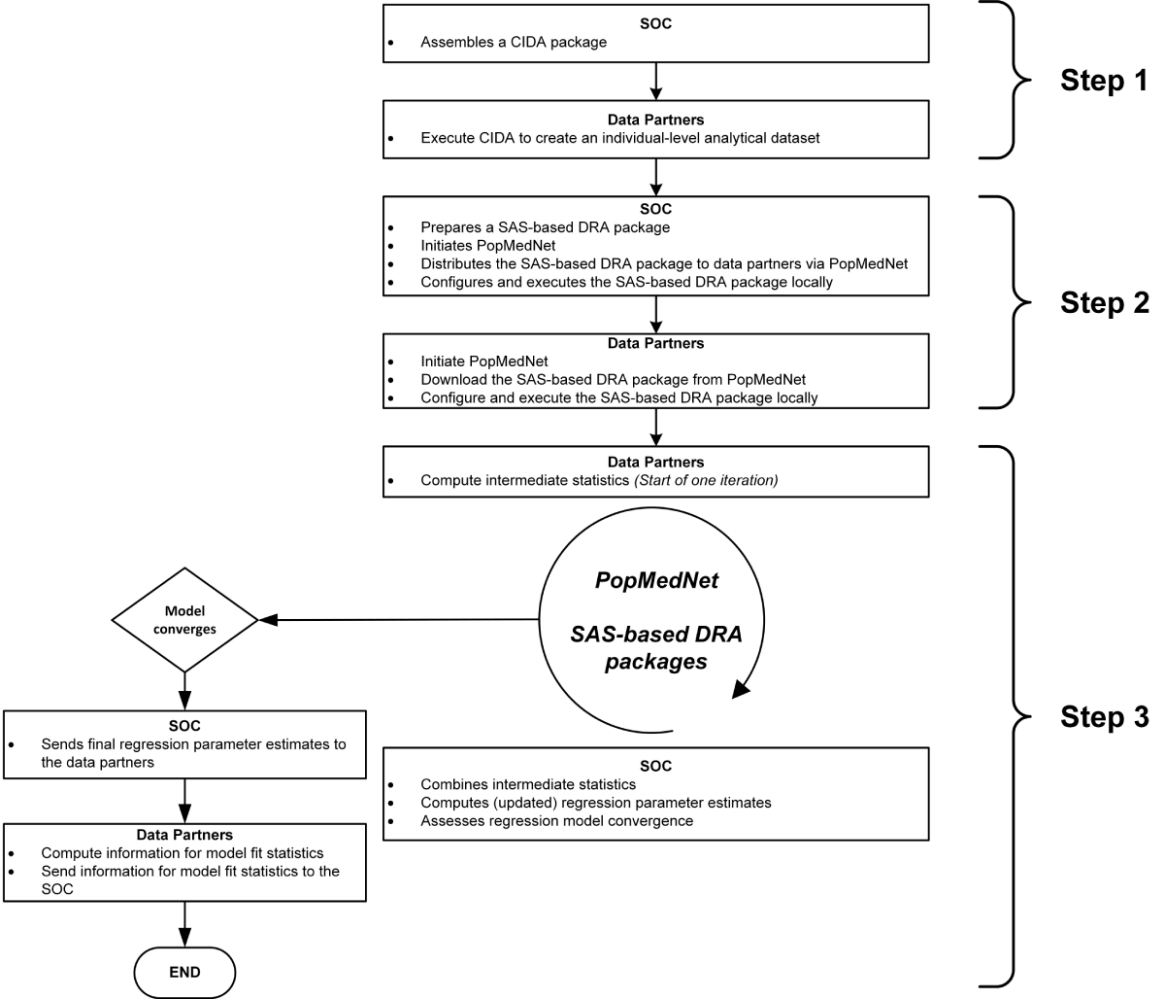
Three-step process to conduct distributed regression analysis with PopMedNet. CIDA: Cohort Identification and Descriptive Analysis Tool; DRA: Distributed Regression Analysis; SOC: Sentinel Operations Center.

A typical DRA includes 3 major steps [3]. Step 1 involves the assembly of a harmonized individual-level analytical dataset at each data partner. In step 2, the analysis center and each data partner execute a DRA algorithm locally. Step 3 involves the iterative transfer of the DRA algorithm outputs between the data partners and the analysis center until the regression model converges or the process reaches a prespecified maximum number of iterations. We used this 3-step process to guide our execution and evaluation of the DRA application with 3 Sentinel data partners, with the Sentinel Operations Center serving as the analysis center (Figure 2).

#### Step 1: Assemble a Harmonized Individual-Level Analytical Dataset at Each Data Partner

We used the CIDA tool (version 3.3.6) to assemble a harmonized individual-level analytical dataset of adult patients aged 18-79 years who received sleeve gastrectomy or Roux-en-Y gastric bypass in any care setting between January 1, 2010 and September 30, 2015 at 3 Sentinel data partners. To be eligible for cohort inclusion, patients must be continuously enrolled in a health plan with medical and drug coverage for at least 1 year before the index bariatric surgery, have at least one weight and height measurement that corresponded to a BMI ≥35 kg/m^2^ in the year before surgery, and have at least one height and weight measurement in the year after surgery. We excluded patients with any bariatric procedure during the 1-year period before the index bariatric surgery. We also excluded patients with gastrointestinal cancer or a revised bariatric surgery procedure on the day of surgery. For each regression analysis, follow-up started on the day of the index bariatric surgery and continued until the occurrence of the outcome of interest (see below), death, end of health plan enrollment, or end of the study period. For distributed linear regression analysis, the outcome was a change in BMI within 1-year postsurgery, defined by subtracting the BMI measurement closest to the end of the 1-year postsurgery date from the last BMI measurement before surgery. For logistic regression, we created a binary outcome variable indicating *1* if the patient had weight loss ≥20% within 1-year postsurgery, and *0* if otherwise. For Cox regression analysis, we computed the time to weight loss ≥20% within the 1-year post-surgery period (Table 1).

**Table 1:** Analytical datasets and variables.

Regression model type	Outcome variable (within 1-year postsurgery)	Variables (exposure and confounders)
Linear	Change in BMI	Bariatric surgery exposure, age at surgery, sex, race and ethnicity, combined Charlson-Elixhauser comorbidity score, number of ambulatory visits, number of other ambulatory visits, number of inpatient stays, number of nonacute institutional stays, number of emergency department visits, BMI before bariatric surgery, number of days between last weight and height measurement and bariatric surgery, and data partner
Logistic	Weight loss ≥20%	Same as above
Cox	Time to weight loss ≥20%	Same as above

#### Step 2: Locally Execute the Distributed Regression Analysis Application at Each Data Partner and the Analysis Center

We assembled 3 separate SAS-based DRA packages to perform distributed linear, logistic, or Cox regression analyses and assessed the association between bariatric procedure (sleeve gastrectomy vs Roux-en-Y gastric bypass) and weight loss within 1-year postsurgery, adjusting for prespecified confounders (Table 1). For Cox regression analysis, we used the Efron approximation to handle tied event times. To be consistent with the standard SAS regression procedures, we prespecified a convergence criterion of <0.01 and a maximum of 25 iterations for distributed logistic and Cox regression analyses.

We distributed each SAS-based DRA package to the 3 data partners through PopMedNet (version 6.7). We instructed the data partners to (1) initiate the automated PopMedNet workflow, allowing the DataMart Client (version 6.7) to automatically download and unzip the SAS-based DRA package to a prespecified local directory, (2) manually place the individual-level analytical dataset created in step 1 in a prespecified local folder*,* (3) specify the file path to the SAS-based DRA package, and (4) execute the SAS-based DRA package in batch mode. Similarly, we instructed the Sentinel Operations Center to (1) initiate the automated PopMedNet workflow, (2) manually place the SAS-based DRA package for the analysis center in a prespecified local directory, (3) specify the file path to the SAS-based DRA package, and (4) execute the SAS-based DRA package in batch mode. Full details of these packages and examples of their execution have been previously described [12,13].

#### Step 3: Iteratively Transfer Distributed Regression Analysis Files Between the Data Partners and the Analysis Center

Once the data partners and the analysis center executed their SAS-based DRA package, the package ran continuously, awaiting input files (eg, updated regression parameter estimates or intermediate statistics) and DRA computation directions (eg, compute intermediate statistics, residuals, and SEs) from the Sentinel Operations Center. We used the PopMedNet workflow to transfer input files and computation directions iteratively and automatically between the data partners and the Sentinel Operations Center.

### Evaluation of Precision and Operational Performance

We requested all data partners to securely transfer their deidentified individual-level analytical datasets to the Sentinel Operations Center. We assessed the precision of the SAS-based DRA package by comparing the DRA parameter estimates and SEs to those obtained from the pooled individual-level data analyses using standard SAS procedures. For distributed linear regression, we compared the model fit statistics *R^2^*, Akaike information criterion (AIC), Sawa’s Bayesian information criterion (BIC), and Schwarz BIC to the statistics obtained from a PROC REG run with the pooled individual-level data. For distributed logistic regression, we compared the model fit statistics log-likelihood, AIC, and Sawa’s BIC to the statistics obtained from a PROC LOGISTIC run with the pooled individual-level data. For distributed Cox proportional hazards regression, we compared the model fit statistics log-likelihood, AIC, and Schwarz BIC to the statistics obtained from a PROC PHREG run with the pooled individual-level data. We considered the integration successful if the DRA parameter estimates and SEs and model fit statistics were precise to the results from the corresponding pooled individual-level data analyses (10^−6^).

For distributed logistic regression, we also compared the receiver operating characteristic (ROC) curve and the area under the ROC curve with the corresponding curve and area obtained from a PROC LOGISTIC run with the pooled individual-level data. We considered the integration successful if the ROC curves were similar in likeliness and if the areas under the curves were comparable. To offer better privacy protection, we summarized individual-level predicted values for the distributed logistic regression analysis in bins of 6. Full details of this approximation method can be found elsewhere [12]. For distributed Cox proportional hazards analysis, we also compared the survival function curve with the curve obtained from a PROC PHREG run with the pooled individual-level data. We considered the integration successful if the survival function curves were similar in likeliness and if the median times to weight loss ≥20% were equivalent.

We extracted time stamps of status changes from PopMedNet and computed the average download, upload, SAS execution, and transfer time at the data partners and the analysis center to evaluate the operational performance of the DRA application. We also reported the average iteration time for each regression model type, and the time required to perform an end-to-end DRA in our test case.

We executed all SAS-based DRA packages in SAS versions 9.3 or 9.4, on a Windows desktop or server routinely used to perform Sentinel queries. All machines used to execute the SAS-based DRA packages and DataMart Client instance operated on a Windows 7 platform, with multiple Intel core processors ranging from 2.3 to 3.4 GHz, and 8 to 16 GB of RAM (Multimedia Appendix 2).

## Results

### Overview

We identified 5452 eligible patients among the 3 participating data partners (n_1_=1706, n_2_=2728, and n_3_=1018). Of these, 981 patients received sleeve gastrectomy, whereas 4471 patients received Roux-en-Y gastric bypass during the study period. Within 1-year postsurgery, the BMI decreased on average by 9.8 kg/m^2^ in sleeve gastrectomy patients and 18.7 kg/m^2^ in Roux-en-Y gastric bypass patients. Five-hundred eighty-two of the 981 (59.3%) patients who had undergone sleeve gastrectomy and 3617 of the 4471 (80.10%) patients who had undergone Roux-en-Y gastric bypass had a weight loss ≥20% within the 1-year postsurgery period. The median time to a weight loss ≥20% was 223.9 days for patients who had undergone sleeve gastrectomy and 196.2 days for patients who had undergone Roux-en-Y gastric bypass.

### Precision

**Figure 3:**
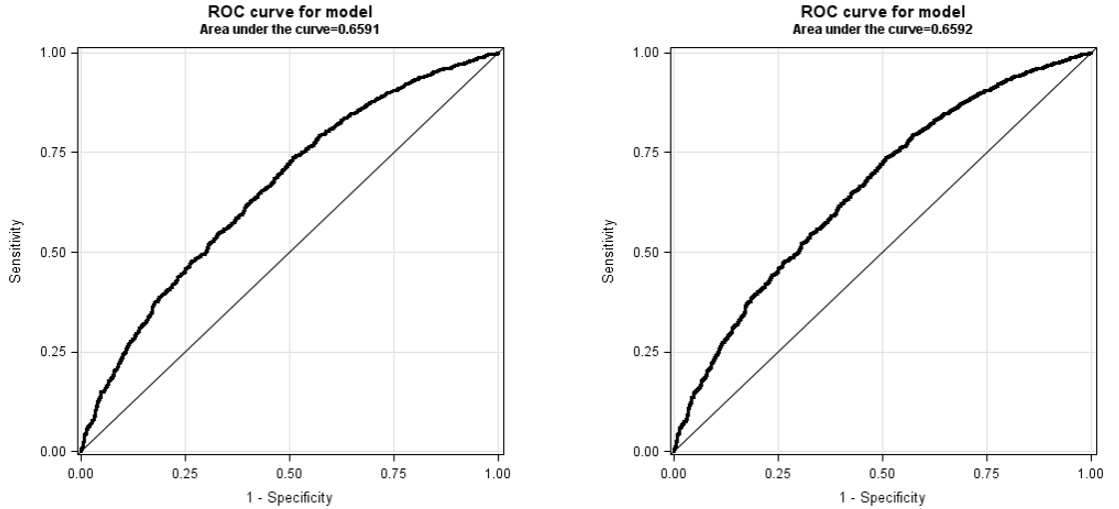
Comparison of receiver operating characteristic curves between distributed logistic regression (left) and pooled individual-level logistic regression (right). To offer better privacy-protecting, individual-level predicted values were summarized in bins of 6 and transferred to the analysis center for aggregation in the distributed logistic regression analysis. The size of the bin is user-specified. ROC: receiver operating characteristic.

**Figure 4:**
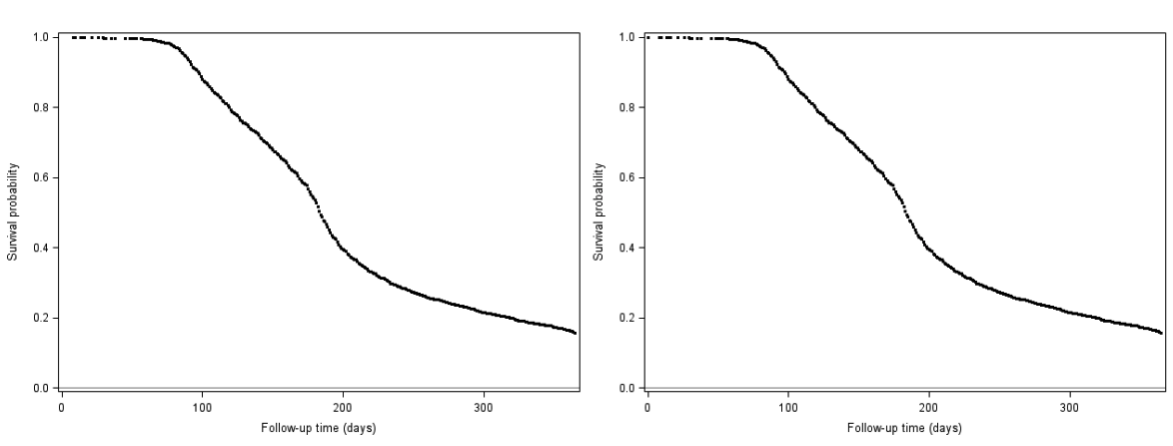
Comparison of survival functions between distributed cox proportional hazards regression (left) and pooled individual-level cox proportional hazards regression (right). The survival curves were evaluated at the mean value of covariates for patients with events.

Tables 2-4 summarize the precision of distributed linear, logistic, and Cox proportional hazards regression analyses. Table 5 shows the model fit statistics of the 3 regression models. All DRA parameter estimates, SEs, and model fit statistics were highly comparable to the estimates obtained from the pooled individual-level analyses that used standard SAS regression procedures. The ROC curve in distributed logistic regression (Figure 3) and the survival function in distributed Cox regression (Figure 4) were similar to those obtained from the pooled individual-level data analyses. The DRA application reported an area under the curve (AUC) of 0.6591 for logistic regression (vs 0.6592 from the pooled individual-level data analysis) and 184 days for Cox proportional hazards analysis (vs 184 days from the pooled individual-level data analysis) as the median time to weight loss ≥20%.

**Table 2:** Distributed linear regression vs pooled individual-level linear regression.

Covariates		Distributed regression analysis		Pooled individual-level analysis			Difference in parameter estimate		Difference in SE
		Parameter estimate	SE	Parameter estimate	SE				
Intercept		34.03935	0.61075	34.03935	0.61075	3.66 x 10^−12^		−9.14 x 10^−13^	
Exposure		2.04714	0.28723	2.04714	0.28723	−4.15 x 10^−^^13^		−4.30 x 10^−^^13^	
Age		−0.03334	0.00837	−0.03334	0.00837	−3.68 x 10^−^^14^		−1.25 x 10^−^^14^	
Preindex BMI		−0.99983	0.00050	−0.99983	0.00050	−6.00 x 10^−^^15^		−7.44 x 10^−^^16^	
Combined comorbidity score		0.04388	0.06949	0.04388	0.06949	3.59 x 10^−^^15^		−1.04 x 10^−^^13^	
Number of ambulatory visits		−0.03068	0.01008	−0.03068	0.01008	−6.59 x 10^−^^17^		−1.51 x 10^−^^14^	
Number of emergency department visits		0.10329	0.08749	0.10329	0.08749	−2.79 x 10^−^^14^		−1.31 x 10^−^^13^	
Number of inpatient visits		0.88725	0.25976	0.88725	0.25976	−6.51 x 10^−^^13^		−3.89 x 10^−^^13^	
Number of nonacute institutional stay		1.32338	1.79056	1.32338	1.79056	4.21 x 10^−^^13^		−2.68 x 10^−^^12^	
Number of other ambulatory visits		0.02159	0.00873	0.02159	0.00873	1.22 x 10^−^^14^		−1.31 x 10^−^^14^	
Days between BMI measurement and index procedure		0.01207	0.00567	0.01207	0.00567	3.92 x 10^−^^15^		−8.48 x 10^−^^15^	
**Race^a^**									
	Unknown	0.94212	0.26841	0.94212	0.26841	−4.16 x 10^−^^13^		−4.02 x 10^−^^13^	
	American Indian or Alaska Native	−0.30948	0.69817	−0.30948	0.69817	−2.39 x 10^−^^13^		−1.04 x 10^−^^12^	
	Asian	−0.16853	0.63001	−0.16853	0.63001	−4.52 x 10^−^^13^		−9.42 x 10^−^^13^	
	Black or African American	1.51961	0.29206	1.51961	0.29206	−9.95 x 10^−^^14^		−4.37 x 10^−^^13^	
	Native Hawaiian or other Pacific Islander	−1.22315	1.04973	−1.22315	1.04973	−4.11 x 10^−^^13^		−1.57 x 10^−^^12^	
Female		−1.22366	0.23205	−1.22366	0.23205	−5.33 x 10^−^^13^		−3.47 x 10^−^^13^	
**Surgery year^a^**									
	2011	0.15150	0.30361	0.15150	0.30361	−5.94 x 10^−^^13^		−4.54 x 10^−^^13^	
	2012	−0.24904	0.30372	−0.24904	0.30372	−6.47 x 10^−^^13^		−4.54 x 10^−^^13^	
	2013	−0.02308	0.30223	−0.02308	0.30223	−6.08 x 10^−^^13^		−4.52 x 10^−^^13^	
	2014	0.32767	0.30609	0.32767	0.30609	−5.93 x 10^−^^13^		−4.58 x 10^−^^13^	
	2015	−0.25767	0.33352	−0.25767	0.33352	−6.18 x 10^−^^13^		−4.99 x 10^−^^13^	
**Data partner site^a^**									
	2	−1.10559	0.31373	−1.10559	0.31373	2.89 x 10^−^^15^		−4.69 x 10^−^^13^	
	3	−0.10990	0.30341	−0.10990	0.30341	−2.07 x 10^−^^13^		−4.54 x 10^−^^13^	

^a^Reference groups: race (white), surgery year (2010), and data partner site (1).

**Table 3:** Distributed logistic regression vs pooled individual-level logistic regression.

Covariates		Distributed regression analysis		Pooled individual-level analysis			Difference in parameter estimate		Difference in SE
		Parameter estimate	SE	Parameter estimate	SE				
Intercept		2.11573	0.22833	2.11573	0.22833	−6.22 x 10^−15^		−1.00 x 10^−14^	
Exposure		1.06711	0.09895	−1.06711	0.09895	−2.00 x 10^−15^		−1.80 x 10^−16^	
Age		−0.01606	0.00316	−0.01607	0.00316	−4.51 x 10^−17^		−1.57 x 10^−16^	
Preindex BMI		0.00003	0.00020	0.00003	0.00020	6.51 x 10^−19^		2.44 x 10^−19^	
Combined comorbidity score		−0.02623	0.02561	−0.02623	0.02561	−6.97 x 10^−16^		−3.12 x 10^−17^	
Number of ambulatory visits		0.01155	0.00447	0.01155	0.00447	6.25 x 10^−17^		1.13 x 10^−17^	
Number of emergency department visits		−0.06230	0.03132	−0.06230	0.03133	3.05 x 10^−16^		1.39 x 10^−17^	
Number of inpatient visits		−0.12098	0.08940	−0.12098	0.08940	1.75 x 10^−15^		−2.36 x 10^−16^	
Number of nonacute institutional stay		0.42510	0.78809	0.42510	0.78809	−2.00 x 10^−15^		−3.33 x 10^−16^	
Number of other ambulatory visits		0.00381	0.00340	0.00381	0.00340	3.17 x 10^−17^		−2.91 x 10^−17^	
Days between BMI measurement and index procedure		−0.00266	0.00201	−0.00266	0.00201	3.90 x 10^−17^		−4.77 x 10^−18^	
**Race^a^**									
	Unknown	−0.39685	0.09485	−0.39685	0.09485	0.00 x 10^+00^		−2.50 x 10^−16^	
	American Indian or Alaska Native	−0.13938	0.26230	−0.13938	0.26230	−1.11 x 10^−16^		5.55 x 10^−17^	
	Asian	−0.37257	0.22341	−0.37257	0.22341	−3.04 x 10^−14^		2.78 x 10^−17^	
	Black or African American	−0.29617	0.10507	−0.29617	0.10507	−3.33 x 10^−16^		−9.71 x 10^−17^	
	Native Hawaiian or Other Pacific Islander	−0.02910	0.40543	−0.02910	0.40543	−6.14 x 10^−16^		0.00 x 10^+00^	
Female		0.19993	0.08422	0.19993	0.08422	−1.80 x 10^−15^		−3.61 x 10^−16^	
**Surgery year^a^**									
	2011	−0.10269	0.11683	−0.10269	0.11684	6.37 x 10^−15^		−5.55 x 10^−17^	
	2012	0.05547	0.11897	0.05547	0.11897	5.45 x 10^−15^		−1.67 x 10^−16^	
	2013	−0.11956	0.11382	−0.11956	0.11382	6.80 x 10^−15^		−1.94 x 10^−16^	
	2014	−0.10956	0.11617	−0.10956	0.11617	4.36 x 10^−15^		−1.80 x 10^−16^	
	2015	0.03701	0.12798	0.03701	0.12798	6.47 x 10^−15^		−2.50 x 10^−16^	
**Data partner site^a^**									
	2	−0.10433	0.11751	−0.10433	0.11751	4.51 x 10^−15^		−9.99 x 10^−16^	
	3	0.75506	0.12577	0.75506	0.12577	2.11 x 10^−15^		−2.50 x 10^−16^	

^a^Reference groups: Race (white), surgery year (2010), and data partner site (1).

**Table 4:** Distributed Cox proportional hazards regression vs pooled individual-level Cox proportional hazards regression.

Covariates		Distributed regression analysis		Pooled individual-level analysis			Difference in parameter estimate		Difference in SE
		Parameter estimate	SE	Parameter estimate	SE				
Exposure		−0.58160	0.05275	−0.58160	0.05275	6.66 x 10^−16^		−8.33 x 10^−17^	
Age		−0.01107	0.00146	−0.01107	0.00146	1.39 x 10^−17^		−9.11 x 10^−18^	
Preindex BMI		−0.00006	0.00009	−0.00006	0.00009	2.85 x 10^−19^		−1.49 x 10^−19^	
Combined comorbidity score		−0.00787	0.01205	−0.00787	0.01205	−3.64 x 10^−17^		−1.04 x 10^−17^	
Number of ambulatory visits		0.00584	0.00158	0.00584	0.00158	−2.95 x 10^−17^		1.08 x 10^−18^	
Number of emergency department visits		−0.01873	0.01679	−0.01873	0.00158	1.56 x 10^−16^		−2.43 x 10^−17^	
Number of inpatient visits		−0.08587	0.04580	−0.08587	0.04580	−9.58 x 10^−16^		−1.25 x 10^−16^	
Number of nonacute institutional stay		0.06626	0.29266	0.06626	0.29266	3.75 x 10^−16^		−3.33 x 10^−16^	
Number of other ambulatory visits		0.00279	0.00134	0.00279	0.00134	4.03 x 10^−17^		−1.52 x 10^−18^	
Days between BMI measurement and index procedure		−0.00221	0.00096	−0.00221	0.00096	2.39 x 10^−17^		−2.17 x 10^−18^	
**Race^a^**									
	Unknown	−0.18898	0.04765	−0.18898	0.04765	5.27 x 10^−16^		0.00 x 10^+00^	
	American Indian or Alaska Native	−0.07476	0.12019	−0.07476	0.12019	1.25 x 10^−16^		2.78 x 10^−17^	
	Asian	−0.22309	0.10933	−0.22309	0.10933	−2.78 x 10^−17^		6.94 x 10^−17^	
	Black or African American	−0.18457	0.05116	−0.18457	0.05116	1.94 x 10^−16^		−1.39 x 10^−17^	
	Native Hawaiian or Other Pacific Islander	−0.19748	0.17333	−0.19748	0.17333	1.42 x 10^−15^		2.78 x 10^−17^	
Female		−0.00887	0.04052	−0.00887	0.04052	−1.24 x 10^−15^		−3.47 x 10^−17^	
**Surgery year^a^**									
	2011	−0.08021	0.05176	−0.08021	0.05176	8.60 x 10^−16^		1.11 x 10^−16^	
	2012	−0.02547	0.05136	−0.02547	0.05136	4.61 x 10^−16^		7.63 x 10^−17^	
	2013	−0.09519	0.05195	−0.09519	0.05195	1.17 x 10^−15^		4.86 x 10^−17^	
	2014	−0.16866	0.05235	−0.16866	0.05235	8.60 x 10^−16^		1.18 x 10^−16^	
	2015	0.24763	0.05640	0.24763	0.05640	3.89 x 10^−16^		1.04 x 10^−16^	
**Data partner site^a^**									
	2	−0.15270	0.05188	−0.15270	0.05188	2.11 x 10^−15^		-6.94 x 10^−18^	
	3	0.33440	0.05161	0.33440	0.05161	8.33 x 10^−16^		2.08 x 10^−17^	

^a^Reference groups: race (white), surgery year (2010), and data partner site (1).

**Table 5:** Comparison of model fit statistics between distributed regression and pooled individual-level data analysis.

Regression model type and statistic or test		Distributed regression analysis	Pooled individual-level data analysis	Difference in model fit statistics
**Linear**				
	*R* ^2^	0.9987	0.9987	3.89 x 10^−^^15^
	Akaike information criterion	20089.6538	20089.6538	−1.59 x 10^−^^08^
	Sawa's Bayesian information criterion	20091.8710	20091.8710	−1.59 x 10^−^^08^
	Schwarz's Bayesian information criterion	20247.5868	20247.5868	−1.59 x 10^−^^08^
**Logistic**				
	-2 log-likelihood	5423.2491	5423.2491	1.36 x 10^−^^11^
	Akaike information criterion	5471.2491	5471.2491	1.36 x 10^−^^11^
	Sawa's Bayesian information criterion	5629.5265	5629.5265	1.36 x 10^−^^11^
	Area under the ROC^a^ curve	0.6591	0.6592	−1.00 x 10^−^^04^
	Hosmer-Lemeshow (chi-square statistics)	1.3405	1.5596	−2.19 x 10^−^^01^
	Hosmer-Lemeshow, *P* value (*df*)	.995 (8)	.991 (8)	3.38 x 10^−^^03^
**Cox**				
	-2 log-likelihood	66217.7270	66217.7270	1.46 x 10^−^^11^
	Akaike information criterion	66263.7270	66263.7270	1.46 x 10^−^^11^
	Schwarz's Bayesian information criterion	66409.6070	66409.6070	1.46 x 10^−^^11^
	Median time to event (days)	184	184	0

^a^ROC: receiver operating characteristic.

### Operational Performance

As expected, the closed-form solution of distributed linear regression analysis required only two iterations, one for computing the regression parameter estimates and SEs and the other for computing the model fit statistics. Both logistic and Cox proportional hazards regression analyses required 6 iterations for model convergence in our test case. Each file transfer process transferred between 3 and 10 files with sizes of 1 to 800 KB.

We extracted 111, 271, and 271 time stamps of status changes from PopMedNet for distributed linear, logistic, and Cox analysis, respectively. Table 6 summarizes the operational performance of the DRA application. It took an average of 102.4 s to complete one DRA iteration across all regression model types. The file transfer workflow (file upload, download, and transfer to the reciprocal party) accounted for 89% of the iteration time. Downloading and uploading the DRA files at the Sentinel Operations Center required an average of 28.6 and 9.8 s, respectively. File transfer from the Sentinel Operations Center to the data partners took on average 9.4 s. Downloading and uploading the DRA files at the data partners required an average of 10.1 and 15.5 s, respectively. File transfer from the data partners to the Sentinel Operations Center took an average 22.1 s. Computing the intermediate statistics at the data partners required an average of 8.0 s, whereas computing the updated regression parameters took an average of 3.8 s at the Sentinel Operations Center.

The distributed Cox regression required the greatest amount of iteration time (113.5 s), followed by logistic regression (95.0 s) and linear regression (91.5 s). Overall, distributed linear regression analysis with our bariatric surgery test case required 440.7 s to complete, whereas logistic and Cox proportional hazards regression analysis required 925.5 and 1016.0 s, respectively.

**Table 6:** Operational performance of the distributed regression analysis application.

Performance metric		Linear	Logistic	Cox	Overall
Required number of iterations for model convergence		2	6	6	—^a^
Total run time		440.7	925.5	1,016.0	—
Average iteration time, mean (SE)		91.5 (10.5)	95 (3.1)	113.5 (5.2)	102.4 (3.8)
**Sentinel operations center (analysis center)**					
	Average download time, mean (SE)	20.5 (5.4)	20.6 (1.3)	39.4 (4)	28.6 (3.2)
	Average computation time, mean (SE)	4.3 (2.6)	3 (1.1)	4.4 (0.4)	3.8 (0.6)
	Average upload time, mean (SE)	8.4 (1.1)	10.2 (0.7)	9.9 (0.6)	9.8 (0.4)
	Average file transfer time (to data partners), mean (SE)	10.5 (0.4)	9.1 (0.5)	9.4 (0.5)	9.4 (0.3)
**Data partners**					
	Average download time, mean (SE)	8.6 (1.2)	10.3 (0.6)	10.3 (0.8)	10.1 (0.4)
	Average computation time, mean (SE)	8.2 (0.8)	7.9 (0.4)	8 (0.3)	8 (0.2)
	Average upload time, mean (SE)	15.6 (1.2)	15.9 (0.6)	15.1 (0.3)	15.5 (0.3)
	Average file transfer time (to analysis center), mean (SE)	20 (0.8)	21.8 (1.9)	23.1 (1.2)	22.1 (1.0)

^a^N/A: not applicable.

## Discussion

### Principal Findings

We have successfully integrated a SAS-based DRA package with PopMedNet, an open-source distributed networking software, and performed DRA in select data partners within a real-world DDN. Our application was able to compute regression parameters, SEs, model fit statistics, and model fit graphics of 3 regression model types (linear, logistic, and Cox proportional hazards) that were within machine precision or similar in likeliness to those produced using standard SAS regression procedures, without the need to share any individual-level data, in under 20 min. The study demonstrated the feasibility and validity of performing multivariable regression analysis in a multicenter setting while limiting the risk of disclosing sensitive individual or institutional information.

### Previous Studies

Previous studies have used simulated or relatively well-controlled distributed environments to demonstrate the ability to perform DRA with only summary-level information [4-8]. These studies have consistently reported that DRA produced precise (generally <10^−12^) results compared with the results from the pooled individual-level data analysis. However, information on the operational performance (computation and file transfer time) of DRA algorithms or workflows is scarce. The closest experience to our DRA application is a Web-based DRA software developed by the SCAlable National Network for Effectiveness Research (SCANNER) [11]. This software is composed of a network portal with a set of Web services and virtual machines that host data from data-contributing sites and several libraries of analytical programs. At the time of our analysis, 3 method libraries were available in the SCANNER software: a cohort discovery tool, an algorithm to perform meta-analyses with distributed data, and an algorithm to perform distributed logistic regression analysis (Grid Binary LOgistic Regression, GLORE) [6]. The authors reported that GLORE produced results equivalent to those from the pooled individual-level data analysis, and software response times of 0.015 s with a dataset of 580 records (with a binary outcome variable, a treatment indicator variable, and 24 covariates) and 27.02 s with a dataset of 10,000 records (with a binary outcome variable and 5 covariates) partitioned among 3 different institutions.

Our DRA application required significantly more time for model convergence than the SCANNER software. However, this additional time for model convergence may be considered marginal in practice, where other aspects of a multicenter study are typically more time-consuming. For example, developing a study protocol and analysis plan or assembling an analytical dataset at each participating data partner for DRA may require considerably more time than the time required to perform DRA. There are also several key differences between our application and the SCANNER software that may explain the difference in operational performance. Specifically, the SCANNER software requires users to install a virtual machine and open ports to the master node hosting the SCANNER hub. This design may have shorter file upload, transfer, and download times between the execution nodes, as files are only transferred between homogeneous virtual machines on the server and not subject to impediments such as firewall security protocols, additional workload, and upload, transfer, and download speeds.

The operational performance of the SCANNER software makes it a desirable option for DRA in networks that are amenable to installing the required software and applications. We previously found that most Sentinel data partners were unwilling to install new software or make modifications to their existing hardware configurations to perform DRA [3]. We chose to develop the DRA application using SAS and PopMedNet because all Sentinel data partners have both software in their systems. In addition, several other large DDNs, including the National Patient-Centered Clinical Research Network [24] and the National Institutes of Health’s Health Care Systems Research Collaboratory [25], use PopMedNet as their file transfer software. In other words, our DRA application requires no new software installation or modifications to existing hardware configurations in DDNs that employ SAS as their statistical software and PopMedNet as their file transfer software. The 3 data partners that participated in this project are also members of numerous PopMedNet-based DDNs. Therefore, the successful integration of our SAS-based DRA package with PopMedNet and execution of DRA with these data partners have the potential to extend DRA beyond the Sentinel System.

### Limitations

Our study is not without limitations. First, DRA requires infrastructure and processes beyond the algorithms and technology described in this paper. For example, DRA with our application requires harmonized individual-level datasets. Since its inception, Sentinel has continuously enhanced its common data model, routine analytical tools, and data quality assurance processes. Thus, Sentinel data partners can rapidly create harmonized analytical datasets for DRA. Research networks and investigators without the same infrastructure may not be able to perform DRA with our application as easily, even if data partners are willing to use PopMedNet as their data-sharing software.

Second, we tested the DRA application with only 3 Sentinel data partners, and all tests were completed in a Windows version of SAS (desktop or server). It is possible that different hardware configurations not found at these data partners or different versions of SAS (Linux or Unix) could change the precision and operational performance or even inhibit the execution of our DRA application. However, we previously found only 3 different configurations of the required hardware components (DataMart Client, SAS software, and the common folder structure) among Sentinel data partners [3]. All 3 hardware configurations were represented among the 3 data partners in this study. We also found the reconfiguration of these components to be relatively straightforward. Therefore, it may be possible to have data partners with other configurations make minor changes to implement our DRA application. During the development of the DRA application, we were able to successfully execute our application on a Linux server with a fourth data partner, by placing the application on a Linux server directory accessible to the DataMart Client as a mapped Windows network drive. This allowed the DataMart Client to access the same file system as the DRA application. Overall, additional testing with more data partners with different hardware configurations and different versions of SAS is needed to fully ensure that our DRA application is operable across different DDNs, research networks, operation systems, and environments.

Third, our precision and operational performances were based on a small sample of successful end-to-end executions of our DRA application. These executions were limited to regression models with 23 variables and analytical datasets ranging from 1000 to 3000 patients distributed among 3 data partners. Future work should include more end-to-end executions, regression models with more variables, datasets of larger sample sizes, and more data partners. However, we found that 89% of the iteration time was attributed to file transfer time, which was largely driven by the number of files, size of the files transferred, and network conditions (upload, download, and transfer speeds, firewall security protocols, and workload). Because the files contain highly summarized information, increasing the number of variables or patients will not increase the number of files or substantially increase the size of the files to be transferred. In this study, each file transfer process transferred files that were less than 1 MB. Our internal testing of analyses with more variables, patients, and data partners did not result in file sizes larger than a few MBs or increased the iteration time. Thus, we do not anticipate DRA with more variables, patients, and data partners in a real-world setting to have a considerable impact on the operational performance of our DRA application. In addition, network conditions at each data partner can vary depending on the workload. We could not vary network conditions at each data partner to formally analyze its impact on the operational performance. However, we did complete our experiments with 3 Sentinel data partners, with machines that are routinely used to fulfill Sentinel query requests. Thus, our results on precision and operational performance likely represent what potential users of DRA will experience in practice.

Fourth, our bariatric surgery test case was relatively simplistic and not as sophisticated as an actual clinical or epidemiologic study. For example, we did not include all the potential confounders. Therefore, the results of our analysis did not have any causal interpretation.

Finally, although DRA uses the intermediate statistics at each data partner to perform multivariable regression analysis, the risk of reidentifying specific individuals is not 0. Under certain conditions (eg, uncommon individual attributes coded with indicator variables), there could be leakage of personal information that could be used to infer or identify specific individuals [26]. To further protect privacy, DRA can be performed using more secure algorithms, such as encrypting or perturbing the intermediate statistics. Future work should explore the integration of these more secure DRA algorithms into our DRA application.

### Conclusions

We have successfully developed and integrated a SAS-based DRA package with an iterative and automatable PopMedNet-driven file transfer workflow to create a DRA application and conduct DRA in select data partners within a real-world DDN. The application produced results that were within machine precision to the results from the pooled individual-level data analyses using standard SAS regression procedures. The end-to-end execution times were reasonable, demonstrating that DRA can be a practical and valid analytical method in real-world settings.

## Appendix

Multimedia Appendix 1 Distributed regression analysis algorithms.

Multimedia Appendix 2 Analysis center and data partner hardware description.

## Abbreviations

AIC: Akaike information criterion
AUC: area under the curve
BIC: Bayesian information criterion
CIDA: Cohort Identification and Descriptive Analysis
DDN: distributed data network
DRA: distribution regression analysis
GLORE: Grid Binary LOgistic Regression
ROC: receiver operating characteristic
SCANNER: SCAlable National Network for Effectiveness Research
