# Supplementary material for: Distributed Regression Analysis Application in Large Distributed Data Networks: Analysis of Precision and Operational Performance
Source: JMIR Med Inform. 2020 Jun 4;8(6):e15073. doi: 10.2196/15073 (PMC7303834; doi:10.2196/15073)
Supplement: Multimedia Appendix 1 [file medinform_v8i6e15073_app1.docx]

**APPENDIX**

# **Appendix A**

# **Distributed regression analysis algorithms**

Regression parameter estimates and standard errors

Linear regression

Let $K$ denote the number of data partners (sites) involved in a distributed regression analysis (DRA), and $n_{k}$ the number of patients at data partner $k=1,\ldots K$. We generalize the structure of the individual-level analytic dataset for DRA at each data partner $k$ as

$$\begin{matrix} w_{1,k} & X_{1,k,1} & \ldots& X_{1,k,p} & Y_{1,k} \\ \vdots& \vdots& \vdots& \vdots& \vdots\\ w_{n_{k},k} & X_{n_{k},k,1} & \ldots& X_{n_{k},k,p} & Y_{n_{k},k} \end{matrix}$$

where $Y_{i,k}$ is the outcome, $\boldsymbol{X}_{\boldsymbol{i,k}}$ a $p* 1$ vector of covariates for individual $i=1,\ldots n_{k}$,and $w_{i,k}$ is an individual-level weight. Let $\boldsymbol{Z}_{i,k}=\mathbf{1||}\boldsymbol{X}_{i,k}$ be the vector of $p+1$ covariates, including the intercept, and $N=\sum_{k=1}^{K} n_{k}$ denote the sum of all observations across data partners.

For linear regression, the regression parameters $\hat{\boldsymbol{\beta}}$ can be estimated using a site-specific sums of squares and cross products ($SSCP$) matrix, which includes all weighted cross products of the variables in the individual-level analytic dataset for site $k$:

| $\boldsymbol{SSSCP}\left( \mathbf{Z}_{k} \vert\vert\boldsymbol{Y}_{k},\mathbf{W}_{k} \right)=\left( \begin{matrix} \sum_{i} {w_{i,k}\boldsymbol{Z}_{\boldsymbol{i,k}}^{\boldsymbol{T}}\mathbf{Z}}_{i,k} & \sum_{i} {w_{i,k}\boldsymbol{Z}_{\boldsymbol{i,k}}^{\boldsymbol{T}}Y}_{i,k} \\ \sum_{i} {{w_{i,k}\mathbf{Z}}_{i,k}Y}_{i,k} & \sum_{i} w_{i,k}Y_{i,k}^{2} \end{matrix} \right)$ | (1) |
| --- | --- |

In SAS, the $\mathrm{SSCP}$ matrix at site $k$ can be computed using the SAS procedure PROC CORR and specifying the SSCP option. The site-specific SSCP matrix from all data partners can be shared with the analysis center and summed to compute a global $\mathrm{SSCP}$ matrix

$$\boldsymbol{SSCP}\left( Z ||Y,W \right)=\sum_{k} \boldsymbol{SSCP}\left( \mathbf{Z}_{k} ||\boldsymbol{Y}_{k},\mathbf{W}_{k} \right)$$

The elements of this global matrix can be used to compute estimates of the linear regression parameters $\hat{\boldsymbol{\beta}}$ and standard errors by inputting this global matrix into the SAS procedure PROC REG and the dataset specified as TYPE=SSCP. In this case, a closed form solution for the regression parameter estimates exists and has the form:

$$\hat{\boldsymbol{\beta}}=\left( \sum_{k=1}^{K} \mathbf{Z}_{k}^{T}\mathbf{W}_{k}\mathbf{Z}_{k} \right)^{-1}\left( \sum_{k=1}^{K} \mathbf{Z}_{k}^{T}\mathbf{W}_{k}\mathbf{Y}_{k} \right)$$

Logistic regression

The above procedure can be extended to accommodate generalized linear models, such as logistic regression models, for which the regression parameter estimates do not have a closed form solution. In this case, estimates of the regression model parameters are obtained via an iteratively reweighted least squares (IRLS) algorithm [1] which, at each iteration $n+1$ solves

$$\sum_{k=1}^{K} \sum_{i=1}^{n_{k}} \tilde{w}_{i,k}(\boldsymbol{\beta}_{n}^{\boldsymbol{T}})\left( \tilde{Y}_{i,k}\left( \boldsymbol{\beta}_{n}^{\boldsymbol{T}} \right)- \boldsymbol{\beta}_{n+1}^{\boldsymbol{T}}\mathbf{Z}_{i,k} \right)\mathbf{Z}_{i,k}=0$$

for $\boldsymbol{\beta}_{n+1}$, where $\boldsymbol{\beta}_{n}$ denotes the estimated regression parameters from the previous iteration,

$\tilde{w}_{i,k}(\boldsymbol{\beta}_{n}^{\boldsymbol{T}})\equiv w_{i,k}\mu^{'}(\boldsymbol{\beta}_{n}^{\boldsymbol{T}}\mathbf{Z}_{i,k})$ denotes a modified weight, $\mu\left( \boldsymbol{\beta}^{T}\mathbf{Z}_{i,k} \right)$ is the expected value of the outcome conditional on the covariates, and $\tilde{Y}_{i,k}\left( \boldsymbol{\beta}_{m}^{\boldsymbol{T}} \right)\equiv\frac{Y_{i,k}-\mu\left( \boldsymbol{\beta}_{n}^{\boldsymbol{T}}\mathbf{Z}_{i,k} \right)}{\mu^{'}\left( \boldsymbol{\beta}_{n}^{\boldsymbol{T}}\mathbf{Z}_{i,k} \right)}+\boldsymbol{\beta}_{n}^{\boldsymbol{T}}\mathbf{Z}_{i,k}$.

The algorithm iterates until a pre-specified convergence criterion is met or a maximum number of iterations is reached. In a distributed setting, this IRLS algorithm can be implemented as follows to obtain the same regression parameter estimates $\hat{\boldsymbol{\beta}}$ and standard errors that would be obtained when individual-level data is pooled across sites:

1. For each iteration $n+1$ at each site $k$, calculate the SSCP matrix

$$\boldsymbol{SSCP}\left( \mathbf{Z}_{k} ||{\tilde{\boldsymbol{Y}}}_{kn}(\boldsymbol{\beta}_{n}),{\tilde{\mathbf{W}}}_{k,n}\boldsymbol{(}\boldsymbol{\beta}_{n}\boldsymbol{)} \right)$$

Bring these SSCP matrices from each site to the analysis center and calculate the combined SSCP matrix as:

| $\boldsymbol{SSCP}\left( \mathbf{Z} \vert\vert{\tilde{\boldsymbol{Y}}}_{n}(\boldsymbol{\beta}_{n}),{\tilde{\mathbf{W}}}_{n}\mathbf{(}\boldsymbol{\beta}_{n}\mathbf{)} \right)=\sum_{k} \boldsymbol{SSCP}\left( \mathbf{Z}_{k} \vert\vert{\tilde{\boldsymbol{Y}}}_{kn}(\boldsymbol{\beta}_{\boldsymbol{n}}),{\tilde{\mathbf{W}}}_{k,n}\boldsymbol{(}\boldsymbol{\beta}_{n} \right)$ | (2) |
| --- | --- |

1. Feed the combined SSCP matrix from Equation (2) into PROC REG to solve for $\boldsymbol{\beta}_{n+1}$
2. Repeat until convergence is achieved. On the iteration $n+1$ that meets the convergence criterion, $\hat{\boldsymbol{\beta}}=\boldsymbol{\beta}_{n+1}$

For convergence criteria we used:

$${\max_{s}|\delta}_{s}^{n+1}|<xconv\_value$$

$$\delta_{s}^{n+1}=\left\{ \begin{aligned} \beta_{s}^{n+1}-\beta_{s}^{n} , \left| \beta_{s}^{n} \right|<0.01 \\ \frac{\beta_{s}^{n+1}-\beta_{s}^{n}}{\beta_{s}^{n}} , else \end{aligned} \right.$$

An additional iteration is required to compute the model goodness-of-fit statistics and statistical tests for the final regression model.

Cox proportional hazards model

For $k=1,\ldots,K$, $m=1,\ldots,M,$ and $i=1,\ldots,N_{m,k}$, let $K$ denote the number of sites, $M$ the number of strata of a specified set of covariates, and $N_{m,k}$ the number of patients at site $k$ in strata $m$. Suppose, among all $N_{m}=\sum_{k=1}^{K} N_{m,k}$ patients in strata $m$, there are $J_{m}$ unique event times, $t_{m,1}<t_{m,2}<\ldots<t_{{m,J}_{m}}$. Denote $(w_{i,m,k},T_{i,m,k},\Delta_{i,m,k},\boldsymbol{Z}_{i,m,k})$ as the observed data for patient $i$ at site $k$ in stratum $m$, with $T_{i,k,m}$ representing the observed follow-up time, $\Delta_{i,m,k}$ the censoring indicator (1 if $T_{i,m,k}$ corresponds to the event time and 0 if the censoring time), $w_{i,m,k}$ an individual-level weight and $\boldsymbol{Z}_{i,m,k}$ a $p*1$ vector of covariates. Define $d_{m,j}=\sum_{k=1}^{K} \sum_{i=1}^{N_{m,k}} I(T_{i,m,k}=t_{m,j},\Delta_{i,m,k}=1)$ as the number of events at time $t_{m,j}$ from all sites. Here the function $I(a=b,c=d,\ldots)$ is defined to be equal to $1$ when all conditions are true and $0$ otherwise.

The input dataset at site $k$ has the following structure for stratum $m$:

| $\begin{matrix} w_{1,m,k} & T_{1,m,k} & Z_{1,m,k,1} & \ldots& Z_{1,m,k,p} & \Delta_{1,m,k} \\ \vdots& \vdots& \vdots& \vdots& \vdots& \vdots\\ w_{N_{m,k},m,k} & T_{N_{m,k},m,k} & Z_{N_{m,k},m,k,1} & \ldots& Z_{N_{m,k},m,k,p} & \Delta_{N_{m,k},m,k} \end{matrix}$ | (3) |
| --- | --- |

Under a stratified Cox model, the hazard function for patients at site $k$ within stratum $m$ for covariate level $\boldsymbol{Z}_{i,m,k}$ is assumed to have the following form:

| $h_{m}\left( t\vert\boldsymbol{Z}_{i,m,k} \right)=\exp\left( \boldsymbol{\beta}^{T}\boldsymbol{Z}_{i,m,k} \right) h_{m}^{(0)}\left( t \right)$ | (4) |
| --- | --- |

where $\boldsymbol{\beta}$ is an unknown $p*1$vector of regression parameter estimates.

We use the Newton-Raphson algorithm to calculate the partial likelihood estimator of the regression parameter estimates $\hat{\boldsymbol{\beta}}$. To apply this algorithm in distributed data networks, the log-likelihood$l\left( \boldsymbol{\beta} \right)$, gradient $\boldsymbol{g}\left( \boldsymbol{\beta} \right)=\frac{\partial l\left( \boldsymbol{\beta} \right)}{\partial\boldsymbol{\beta}}$, and the Hessian matrix $\boldsymbol{H}\left( \boldsymbol{\beta} \right)=\frac{\partial^{2}l\left( \boldsymbol{\beta} \right)}{\partial\boldsymbol{\beta}\partial\boldsymbol{\beta}^{T}}$ must be expressed in terms of aggregated quantities from each data partner. Let’s first define the quantities that have to be calculated at each site $k$ in each stratum $m$.

Define the following:

$$l_{m}\left( \boldsymbol{\beta} \right)=\sum_{j} \left\{ \boldsymbol{\beta}^{T}\boldsymbol{d}_{m,j}^{\left( \boldsymbol{1} \right)} -d_{m,j}^{\left( 0 \right)}\log S_{m,j}^{\left( 0 \right)}\left( \boldsymbol{\beta} \right) \right\}$$

$$\boldsymbol{g}_{m}\left( \boldsymbol{\beta} \right)= \sum_{j} \left\{ {\boldsymbol{d}_{m,j}^{\left( \boldsymbol{1} \right)}-d}_{m,j}^{\left( 0 \right)} \frac{\boldsymbol{S}_{m,j}^{\left( 1 \right)}\left( \boldsymbol{\beta} \right)}{S_{m,j}^{\left( 0 \right)}\left( \boldsymbol{\beta} \right)} \right\}$$

$$\boldsymbol{H}_{m}\left( \boldsymbol{\beta} \right)=-\sum_{j} d_{m,j}^{(0)} \left\{ \frac{\boldsymbol{S}_{m,j}^{\boldsymbol{(}2\boldsymbol{)}}(\boldsymbol{\beta)}}{S_{m,j}^{(0)}\left( \boldsymbol{\beta} \right)}-\frac{\boldsymbol{S}_{m,j}^{\boldsymbol{(}1\boldsymbol{)}}(\boldsymbol{\beta)}*\left[ \boldsymbol{S}_{m,j}^{\boldsymbol{(}1\boldsymbol{)}}(\boldsymbol{\beta)} \right]^{\boldsymbol{T}}}{\left[ S_{m,j}^{(0)}\left( \boldsymbol{\beta} \right) \right]^{2}} \right\}$$

$$\boldsymbol{d}_{m,j,k}^{\left( l \right)}=\sum_{i=1}^{N_{m,k}} I\left( T_{i,m,k}=t_{m,j},\Delta_{i,m,k}=1 \right)w_{i,m,k}\boldsymbol{Z}_{i,m,k}^{l}$$

$$\boldsymbol{S}_{m,j,k}^{\left( l \right)}\left( \boldsymbol{\beta} \right)=\sum_{i=1}^{N_{m,k}} I\left( T_{i,m,k}\geq t_{m,j} \right)w_{i,m,k}\exp\left( \boldsymbol{\beta}^{T}\boldsymbol{Z}_{i,m,k} \right)\boldsymbol{Z}_{i,m,k}^{l}$$

We use a notation in which an absence of an index in a matrix implies summation over that index. For example,

| $\boldsymbol{d}_{m,j}^{\boldsymbol{(}l\boldsymbol{)}} \boldsymbol{=}\sum_{k} \boldsymbol{d}_{m,j,k}^{\boldsymbol{(}l\boldsymbol{)}}$ | $\boldsymbol{S}_{m,j}^{(l)}=\sum_{k} \boldsymbol{S}_{m,j,k}^{(l)}$ | (5) |
| --- | --- | --- |

| $l\left( \boldsymbol{\beta} \right)=\sum_{m=1}^{M} l_{m}\left( \boldsymbol{\beta} \right)$ | $\boldsymbol{g}\left( \boldsymbol{\beta} \right)=\sum_{m=1}^{M} \boldsymbol{g}_{m}\left( \boldsymbol{\beta} \right)$ | $\boldsymbol{H}\left( \boldsymbol{\beta} \right)=\sum_{m=1}^{M} \boldsymbol{H}_{m}\left( \boldsymbol{\beta} \right)$ | (6) |
| --- | --- | --- | --- |

The summarized matrices $\boldsymbol{d}_{m,j,k}^{\left( l \right)}$ and $\boldsymbol{S}_{m,j,k}^{\left( l \right)}$ are computed at each data partner and only these are shared with the analysis center to produce regression parameter estimates and standard errors. Like logistic regression, a closed form solution for the regression parameter estimates in this case does not exist. The partial likelihood estimator of $\beta$ is obtained by the Newton-Raphson algorithm, which on each iteration $n$ solves:

| $\boldsymbol{-H}\left( \boldsymbol{\beta}_{n} \right)\left( \boldsymbol{\beta}_{n+1}\boldsymbol{-}\boldsymbol{\beta}_{n} \right)\boldsymbol{=g}\left( \boldsymbol{\beta}_{n} \right)$ |  |
| --- | --- |

for $\boldsymbol{\beta}_{n+1}$**.** The algorithm iterates until a pre-specified convergence criterion is met or a maximum number of iterations is reached.

To implement this algorithm at the analysis center based only on the highly summarized gradient and Hessian matrices, we use SAS PROC REG to solve a system of linear equations. Specifically, we construct a SSCP dataset as follows

$$\boldsymbol{SSCP}=\left( \begin{matrix} \boldsymbol{-H}\left( \boldsymbol{\beta} \right) & {\boldsymbol{g}\left( \boldsymbol{\beta} \right)}^{\mathbf{T}} \\ \boldsymbol{g}\left( \boldsymbol{\beta} \right) & const \end{matrix} \right)$$

and feed it into PROC REG as an input dataset with the property TYPE=SSCP. This produces output$\boldsymbol{b}_{n} =\boldsymbol{\beta}_{n+1}-\boldsymbol{\beta}_{n}=\boldsymbol{H}^{\boldsymbol{-1}}\left( \boldsymbol{\beta}_{n} \right)\boldsymbol{g}\left( \boldsymbol{\beta}_{n} \right)$, from which we can obtain $\boldsymbol{\beta}_{n+1}$ by

$$\boldsymbol{\beta}_{n+1}\boldsymbol{=}\boldsymbol{\beta}_{n}\boldsymbol{-}\boldsymbol{H}^{\boldsymbol{-1}}\left( \boldsymbol{\beta}_{n} \right)\boldsymbol{g}\left( \boldsymbol{\beta}_{n} \right)$$

PROC REG also outputs the inverse, $\mathbf{I}^{\boldsymbol{-1}}$of the matrix $\mathbf{I}\left( \boldsymbol{\beta} \right)=-\boldsymbol{H}=-\frac{\partial^{2}l\left( \boldsymbol{\beta} \right)}{\partial\boldsymbol{\beta}\partial\boldsymbol{\beta}^{T}}$ evaluated at the final partial likelihood estimate $\boldsymbol{=}\hat{\boldsymbol{\beta}}$ . This gives us the estimated covariance matrix:

| $\hat{cov}\left( \hat{\boldsymbol{\beta}} \right)\mathbf{=}\mathbf{I}^{\mathbf{-1}}\left( \hat{\boldsymbol{\beta}} \right)$ | (7) |
| --- | --- |

Regression model goodness-of-fit measures and statistical tests

Some goodness-of-fit measures, graphics, and statistical tests for linear, logistic, and Cox proportional hazards regression models can be computed without sharing any individual-level data. Site-specific components for measures such as $R^{2}$, Akaike Information Criterion (AIC), Sawa’s Bayesian Information Criterion (SBC), Schwarz’s Bayesian Criterion (BIC), and the log-likelihood can be computed at each data partner and shared with the analysis center for aggregation. Goodness-of-fit graphics, measures, and tests such as a receiver operating characteristic (ROC) curve, area under the ROC curve (AUC), and Hosmer-Lemeshow statistics and p-value for logistic regression require individual-level predicted values. To protect privacy, we used an approximation approach to summarize individual-level predicted values in bins and transferred to the analysis center for aggregation. Full details and an example computation of these statistics and tests have been previously described [2, 3].

# **REFERENCE**

1. McCullagh P, Nelder JA. *Generalized Linear Models, Second Edition*: Taylor & Francis, 1989.

2. Her QL, Vilk Y, Young J, et al. A distributed regression analysis application based on SAS software. Part I: Linear and logistic regression. ArXiv e-prints 2018. <https://ui.adsabs.harvard.edu/#abs/2018arXiv180802387H> (accessed April 15, 2019).

3. Vilk Y, Zhang Z, Young J, et al. A distributed regression analysis application based on SAS software Part II: Cox proportional hazards regression. ArXiv e-prints 2018. <https://ui.adsabs.harvard.edu/#abs/2018arXiv180802392V> (accessed April 15, 2019).
