# Supplementary material for: Distributed Regression Analysis Application in Large Distributed Data Networks: Analysis of Precision and Operational Performance
Source: JMIR Med Inform. 2020 Jun 4;8(6):e15073. doi: 10.2196/15073 (PMC7303834; doi:10.2196/15073)
Supplement: Multimedia Appendix 2 [file medinform_v8i6e15073_app2.docx]

**APPENDIX**

**Appendix B**

| **Table B1: Analysis Center and Data Partner Hardware Description** | | | |
| --- | --- | --- | --- |
| **Site** | **Operating System** | **Processor** | **Random Access Memory** |
| Sentinel Operations Center | Microsoft Windows 7 Professional | Intel(R) Core(TM) i5-6300U CPU @ 2.40GHz, 2401 Mhz, 2 Cores, 4 Logical Processors | 8 GB |
| Data Partner 1 | Microsoft Windows 7 Professional | Intel(R) Core(TM) i7-4810MQ CPU @ 2.80GHz, 3401 Mhz, 4 Cores, 8 Logical Processors | 16 GB |
| Data Partner 2 | Microsoft Windows 7 Professional | Intel(R) Core(TM) i7-6700 CPU @ 3.40GHz, 3401 Mhz, 4 Cores, 8 Logical Processors | 16 GB |
| Data Partner 3 | Microsoft Windows 7 Enterprise | Intel(R) Core(TM) i7-6700 CPU @ 3.40GHz, 3401 Mhz, 4 Cores, 8 Logical Processors | 16 GB |
